# Preliminary study on biology and feeding capacity of *Chelisoches morio* (Fabricius) (Dermaptera:Chelisochidae) on *Tirathaba rufivena* (Walker)

**DOI:** 10.1186/s40064-016-3628-9

**Published:** 2016-11-09

**Authors:** Baozhu Zhong, Chaojun Lv, Weiquan Qin

**Affiliations:** Hainan Key Laboratory of Tropical Oil Crop Biology/Coconut Research Institute, Chinese Academy of Tropical Agricultural Sciences, Wenchang, 571339 Hainan People’s Republic of China

**Keywords:** *Chelisoches morio*, Biology, Predator, *Tirathaba rufivena*, Feeding capacity

## Abstract

**Background:**

*Chelisoches morio* (Fabricius) (Dermaptera:Chelisochidae) is an important predator of *Tirathaba rufivena* (Walker) (Lepidoptera:Pyralidae). For better use of the natural enemy, a biological study on *C. morio* was conducted, particularly its developmental duration, survival, fecundity and sex ratio. And the feeding capacity of *C. morio* against *T. rufivena* was also studied under laboratory conditions.

**Results:**

The biological study on *C. morio* was revealed that female adults usually lay eggs in egg masses. The number of eggs per female averaged 140.17 eggs, and the incubation period was 7.50 days. The duration of nymphal development included four instars. The immature period was 83.95 days. The longevity of males and females was 58.60 and 93.55 days, respectively. The results from the study on the feeding capacity of *C. morio* using *T. rufivena* as food revealed that *C. morio* adults could consume 11.08, 7.87, 7.09, 6.82 and 5.89 first-to-fifth-instar *T. rufivena* larvae, respectively.

**Conclusions:**

*C. morio* showed a huge amount of predation on this pest at all larval stages, implying a significant potential for the use of *C. morio* in controlling *T. rufivena*.

## Background

*Tirathaba rufivena* is a moth of the Pyralidae family. It is a major pest insect of palm plants such as *Areca catechu* L. (betel), *Cocos nucifera* L. (coconut), and *Elaeis guineensis* Jacq. It is found from Southeast Asia to the Pacific Islands, including China, Malaysia, the Cook Islands, the Philippines and the tropical region of Queensland. In Hainan, China, damage to palm plants by *T. rufivena* has been severe and widespread. This pest has significantly affected the production of palm plants; the damage rate was found to be 10–67% in betel plants (*A. catechu* L.) and 10–40% in betel blooms, fruit, and fringe (Fan et al. 1986, 1991). Presently, the control of *T. rufivena* is still focused on chemical pesticides, and this could lead to many problems, for example, resistance to traditional insecticides, environmental pollution, human health impacts, and injury to beneficial species.

Biological control is often considered to be one of the most efficient technique in integrated pest management, and the parasitoid *Elasmus punctulatus* Verma Hayat was found by Gan et al. (2010, 2011) have a controlling effect on this pest, with a parasitism rate of 20–30% in the field. The biology and mass rearing of this parasitoid in an insectary was then studied. However, parasitic wasps are vulnerable to climate and require a long time for effective control to occur, leading to instability in their controlling effect. Predators, such as *Formica* spp., *Sitticus* spp., *Lycosa* spp., *Forcipula* spp., Coccinellidae, and others, are another kind of natural enemy in the control of pests, which has been demonstrated by previous studies (Hollingsworth et al. 1988; Zeng et al. 2004; Luo et al. 2005; Wu et al. 2006; Zou et al. 2008; Zhang et al. 2009). The black earwig, *Chelisoches morio* (Fabricius) is an important predator of many insect pests. Li et al. (2011) reported that *C. morio* adults can preys upon different stages of *Brontispa longissima* (Coleoptera:Hispidae), and has been used in field for preventing the coconut leaf beetle *B. longissima* in Thailand and Philippins (Chomphukhieo et al. 2008). Abraham and Kurian (1974) showed that *C. morio* predates eggs and young larvae of red palm weevil *Rhynchophorus ferrugineus* (Oliv.) (Curculionidae:Coleoptera). Zhong et al. (2016) indicated that *C. morio* is one of the predominant predators showing the potential to control *T. rufivena*. However, few studies have presented results regarding its biology and feeding capacity on *T. rufivena*.

This study investigates the biology of *C. morio*, particularly its developmental duration, survival, fecundity and sex ratio. The feeding capacity of *C. morio* against *T. rufivena* was also studied under laboratory conditions.

## Results

### Morphological characteristics

The abdomens of *C. morio* male and female adults connect when mating, and the mating time varies from 2 to 40 min. Females can perform multiple matings. Female adults begin to lay eggs 4–6 days after mating, and the eggs are generally deposited in masses of 35.7 individuals, on average. In total, the females lay an average of 140.17 eggs each. The adults often flip the eggs after spawning; if outside interference is encountered, the eggs will be transferred to a new hidden area. Newly spawned eggs are creamy white and then turn white and clear when they are near hatching, after 7–8 days. The average width and length are 0.75 and 1.03 mm, respectively. The nymphs have 4 instars, and the newly hatched nymphs are milky white with limited activity and need to be fed by adults. The nymphs can self-feed after a molt. The second- and third-instar larvae are similar, differing only in body length.

The adult body is shiny black or dark brown and has a long and narrow shape with forceps. The forceps of the male adult are longer, and the edge exhibits multiple serrations, which was not obviously seen in females.

### The developmental duration of the immature stages

The developmental duration for the eggs of *C. morio* was 7.50 days, and that of first- to fourth-instar nymphs was 12.75, 10.63, 14.53 and 21.89 days, respectively, under the conditions of rearing with areca core leaves at a constant temperature (26 ± 1 °C) and a relative humidity (RH) of 65 ± 5%. Subsequently, the total developmental period (from egg to adult emergence) was 83.95 days (Table 1).

**Table 1:** Duration period and survivor of various developmental stages of *C. morio* Fabricius under laboratory condition (26 ± 1 °C and 65 ± 5% RH)

Stage of development	N	Mean ± SE (days)	Range (days)	Survival rate (%)
Egg	20	7.50 ± 0.11	7–8	100
Nymph				
Instar I	20	12.75 ± 0.10	12–13	100
Instar II	19	10.63 ± 0.16	10–12	99
Instar III	19	14.53 ± 0.14	13–15	99
Instar IV	19	21.89 ± 0.20	20–23	99
Immature^a^	19	83.95 ± 0.97	78–90	99

^a^Immature refers to the developmental duration of *C. morio* from egg to adult emergence

The longevity of *C. morio* adults was observed under laboratory conditions. The life span of adult male and females supplemented with honey water for nutrition was an average of 58.60 ± 0.52 and 93.55 ± 1.25 days, respectively. The fecundity of female *C. morio* was 140.17 ± 0.44 on average.

### Feeding capacity of *C. morio* against *T. rufivena* larvae

The daily predation of first-to-fifth-instar larvae of *T. rufivena* by *C. morio* was 11.08, 7.87, 7.09, 6.82 and 5.89, respectively. The maximum feeding capacity of *C. morio* was observed for first-instar larvae of *T. rufivena*, and the minimum predation was observed against fifth-instar larvae. The results showed that the consumption of prey by *C. morio* adults decreased as the age of the prey increased (Table 2).

**Table 2:** Feeding capacity of *C. morio* (Fabricius) against *T. rufivena* under laboratory condition (26 ± 1 °C and 65 ± 5% RH)

Stage of nymph development	Replicates	Mean ± SE (days)	Range (days)
Instar I	15	11.08^a^ ± 0.15 a	9–13
Instar II	15	7.87 ± 0.09 b	7–9
Instar III	15	7.09 ± 0.12 c	6–9
Instar IV	15	6.82 ± 0.72 c	5–8
Instar V	15	5.89 ± 0.71 d	5–7

^a^Means bearing the same letters are not significantly different (LSD; *P* < 0.05) between treatments

## Discussion

Biology is the basis of the study of insects. Understanding the life history of insects is the basis of mass rearing insect natural enemies in an insectary. The biology of the black earwig, *C. morio*, was reported overseas many years ago, but there are some differences compared to the results of this study, which may be a result of differences in the geographical environment, climate, indoor rearing conditions and the influences of different foods. Using *B. longissima* (Coleoptera:Hispidae) as food, Chomphukhieo et al. (2008) reported that the longevity of male and female adults was 51.40 ± 0.89 and 76.75 ± 1.28 days, respectively, while females and males can live 93.55 and 58.60 days, respectively, when provided with honey water as supplementary nutrition. Abraham and Kurian (1974) indicated that females feeding with red palm weevil eggs and young larvae lay an average of 156 eggs each, while 140.17 eggs were observed in our study.

The newly hatched nymphs were raised by *C. morio* adults, indicating that the small nymphs will have difficulty surviving without adult parenting, which in accordance with the research of Lv et al. (2012). In addition, we also observed that eggs cannot hatch if the eggs and adults are separated. Wang (1979) and Lv et al. (2012) showed that, in the hatching process, female earwig adults not only keep the eggs at a certain temperature but also that female adults often use their mouthparts to flip the eggs to facilitate egg heating and effective incubation. The understanding of these special habits of *C. morio* may provide help and reference for large-scale indoor breeding efforts.

The black earwig, *C. morio*, not only preys upon different stages of *B. longissima* (Chomphukhieo et al. 2008; Li et al. 2011; Lv et al. 2012) but also predates red palm weevil eggs and young larvae (Abraham and Kurian 1974). Our study indicated that *C. morio* adults were able to predate *T. rufivena* larvae, especially younger and smaller larvae (11.08), but that they had poorer ability to feed on the old larvae, based on the number predated (5.89). This difference may be related to the insect body nutrient content and the fact that larvae spin silken webs; the nutrition provided by small larvae is limited, while the nutrient content of larger larvae is high but they engage in more spinning, which could restrict black earwig activity and affect their search for prey.

The black earwig, *C. morio*, is a natural enemy with high activity, and the author survey found that the black earwig prefers a dark and humid habitat. *C. morio* has potential predator characteristics, as it is often hidden in the axils of leaves or flowers near pests feeding on the leaves and flowers of palm plants, and this species can be used as a supplementary measure together with parasitic wasps in the prevention and control of *T. rufivena*.

## Conclusions

In summary, *C. morio* (Dermaptera:Chelisochidae) is an important predator of *T. rufivena* Walker, and the biological characteristics of this predator were investigated in this study. *C. morio* showed a huge amount of predation on this pest at all larval stages, implying a significant potential for the use of *C. morio* in controlling *T. rufivena.*

## Methods

### Predator and prey

A laboratory population of *T. rufivena* was established by collecting larvae from infested betel field. The larvae were reared with areca core leaves at a constant temperature (26 ± 1 °C), 65 ± 5% RH and a photoperiod of 0:24 L:D in the laboratory, Department of Plant Protection, Coconut Research Institute, Chinese Academy of Tropical Agricultural Sciences, Wenchang, Hainan Province. Individuals of *C. morio* were also collected from the betel field and maintained on *T. rufivena* larvae for more than ten generations under the same laboratory conditions before they were used in this study. Adults of *C. morio* were supplemented with honey water for nutrition.

### Observation of biological characteristics of *C. morio*

This study was conducted under laboratory conditions at a temperature of 26 ± 1 °C, 65 ± 5% RH and a photoperiod of 0:24 L:D. Second-instar larvae of *T. rufivena* were placed into a clean transparent glass tube (20 mm × 200 mm), and a pair of newly emerged (one female and one male) *C. morio* adults was then introduced. Areca core leaves were placed in the glass tube for *T. rufivena* feeding, and the tube was covered with gauze. Additional larvae were added as they were eaten by *C. morio*. Biological characteristics were studied by daily observation of *C. morio*, including adult mating and spawning behavior and the habits of various developmental stages. This study was repeated 20 times.

### Feeding capacity of *C. morio* against *T. rufivena* larvae

Healthy first-, second-, third-, fourth-, and fifth-instar *T. rufivena* larvae were placed into clean transparent plastic boxes (10 cm × 5 cm × 5 cm) with areca core leaves. Each box contained 15 larvae, and 1 female adult black earwig that had been starved for 24 h was introduced. The number of predated larvae was assessed and recorded daily. Continuous observation occurred for 3 days, and larvae were added according to the number predated. This experiment was repeated 15 times.

### Data analysis

The data were analyzed by SPSS 13.0 software, and the results were expressed as the mean ± standard error (mean ± SE).

## Abbreviations

*C. morio*: *Chelisoches morio*
*T. rufivena*: *Tirathaba rufivena*
*B. longissima*: *Brontispa longissima*
RH: a relative humidity
